# An efficient learning based approach for automatic record deduplication with benchmark datasets

**DOI:** 10.1038/s41598-024-63242-1

**Published:** 2024-07-15

**Authors:** M Ravikanth, Sampath Korra, Gowtham Mamidisetti, Maganti Goutham, T. Bhaskar

**Affiliations:** 1 Department of CSE, Malla Reddy University, Maisammaguda, Kompally, Hyderabad, India; 2 Department of CSE, Sri Indu College of Engineering and Technology (A), Sheriguda, Ibrahimpatnam, Hyderabad, T.S 501510 India; 3 Department of CSE, Malla Reddy University, Maisammaguda, Kompally, Hyderabad, 500100 India; 4 Department of CSE CMR College of Engineering and Technology, Kandlakoya, Medchal, Hyderabad, TS 50140 India

**Keywords:** Record deduplication, Deep learning, Word embeddings, Long short term memory, Data integration, Engineering, Physics

## Abstract

With technological innovations, enterprises in the real world are managing every iota of data as it can be mined to derive business intelligence (BI). However, when data comes from multiple sources, it may result in duplicate records. As data is given paramount importance, it is also significant to eliminate duplicate entities towards data integration, performance and resource optimization. To realize reliable systems for record deduplication, late, deep learning could offer exciting provisions with a learning-based approach. Deep ER is one of the deep learning-based methods used recently for dealing with the elimination of duplicates in structured data. Using it as a reference model, in this paper, we propose a framework known as Enhanced Deep Learning-based Record Deduplication (EDL-RD) for improving performance further. Towards this end, we exploited a variant of Long Short Term Memory (LSTM) along with various attribute compositions, similarity metrics, and numerical and null value resolution. We proposed an algorithm known as Efficient Learning based Record Deduplication (ELbRD). The algorithm extends the reference model with the aforementioned enhancements. An empirical study has revealed that the proposed framework with extensions outperforms existing methods.

## Introduction

Record deduplication has attracted many researchers due to the exponential growth of textual content in the cloud and other storage platforms. Moreover, organizations are heavily exploring the mining of data to arrive at business intelligence for making expert decisions. Of late machine learning and deep learning models assumed significance due to their learning capabilities and efficiency in entity resolution. Machine learning techniques explored in^1–4^ and^5^ revealed the significance of learning-based approaches in resolution of duplicate entities in real-world datasets. ML techniques consider this problem as binary classification. Two relational tables such as A and B can have duplicate records that lead to wastage of resources, resource wastage and data inconsistencies. Therefore, it is essential to eliminate such duplicates and the process of identification and removal of duplicates is known as deduplication. It finds all matching records and classifies them considering 1 for a match and 0 for a non-match. In the process of finding similarities between entities or records, it is important to make use of similarity measures. Various ML models such as Support Vector Machines (SVM) are used widely for this kind of research. There are ensemble methods as well. Ensemble methods exploit the knowledge of multiple models and a voting kind of method is used to arrive at the final binary outcome of the classification. There are many deep learning methods found in the literature for the efficient removal of duplicate records as explored in^6^ and^7^ to mention a few. Deep learning models are advanced neural network-based methods that have the potential to deal with in-depth learning processes that could render useful knowledge to those models. With a training-based approach, the deep learning models could outperform traditional models. Many researchers contributed to building deduplication frameworks for the efficient detection of duplicate records. They include Bayeswipe^8^, Dedoop^9^, Eraser^10^, r-HUMO^2^ which is a risk-aware model and SEM + ^11^.

From the literature, it was observed that ML and deep learning models are used for deduplication besides heuristics-based approaches. Particularly, we found the model in^6^ is based on deep learning and is efficient. However, it has specific limitations such as a lack of support for numeric attributes, null values and trainable embeddings besides the usage of multiple combinations of similarity measures. These shortcomings of^6^ are overcome in this paper by proposing extensions to the baseline models presented. Therefore, we considered the model in^6^ as a reference model for improvements. Therefore, the main objective of this research is to enhance the model presented in^6^ with extensions to overcome the aforementioned problems. Our contributions are as follows.

1. We proposed a framework known as Enhanced Deep Learning-based Record Deduplication (EDL-RD) for improving the performance of a reference or baseline model explored in^6^.
2. We proposed an algorithm known as Efficient Learning based Record Deduplication (ELbRD). The algorithm extends the reference model with different enhancements such as multiple similarity functions, support for numeric attributes, null values and trainable embeddings.
3. We extended the reference model’s implementation with the aforementioned extensions and evaluated the same. Our empirical results revealed that the proposed extensions to the reference model outperform the variants of the reference model provided in^6^.

The remainder of the paper is structured as follows. Section "Related work" reviews the literature on prior works extensively to ascertain the merits and demerits of the state-of-the-art models. Section "Preliminaries" provides the required preliminary details to easily understand the proposed work in this paper. Section "Methodology" presents the reference model along with proposed extensions to it. Section "Experimental setup" presents the experimental setup. Section "Results and discussion" provides details about the practical and managerial implications of the proposed methodology. Section "Conclusion and future work" provides the results of our experiments while Section 8 throws light on concluding and the future scope of the research.

## Related work

This section reviews prior works on record deduplication. It throws light on various kinds of methods including ML-based approaches. García-Gil et al.^12^ focused on smart data concepts and in the process, they proposed a noise-filtering approach to eliminate inconsistencies in big data before its classification. Chen et al.^13^ focused on graph embeddings consisting of multiple language data for cross-lingual knowledge management. De et al.^8^ proposed a framework named Bayeswipe for automatically detecting duplicates and improving data quality. Guo et al.^14^ exploited knowledge graphs to discover long-term dependencies among entities. Their work could find inconsistencies in data towards improving the quality of the data. In^6^ there is a deduplication method based on deep learning and its variants. However, this could be improved with different optimizations. In this paper, we used it as a reference model and proposed various extensions to it. Kolb et al.^9^ proposed a framework for efficient deduplication. Their framework was named Dedoop which is implemented using a distributed framework known as Hadoop. Eraser is another framework developed by Mayfield et al.^10^ for cleaning duplicates in the given data which is in a structured format. It makes use of statistical inference. Li et al.^15^ proposed a model for entity resolution and alignment with the help of a cross-graph model and knowledge embedding model. Zhu et al.^16^ focused on word embeddings with a notion of joint knowledge embeddings and entity alignment with an iterative process. Hou et al.^2^ proposed a framework known as r-HUMO which is risk-aware for entity resolution and provides quality guarantees.

Papadakis et al.^17^ proposed different techniques based on filtering and blocking techniques to achieve efficient resolution of entities. Kong et al.^18^ proposed a methodology for checking entity matches from data sources obtained from heterogeneous environments. Efthymiou et al.^19^ focused on blocking algorithms and their benchmarking towards web entity resolution. Their method was found efficient when compared with the state of the art. Fan et al.^3^ proposed a method for enhancing the quality of entities using machine-learning approaches combined with logic rules. Köpcke et al.^20^ explored different entity resolution methods in terms of their merits and shortcomings. Wu et al.^21^ investigated the entity alignment and joint learning of entities. Their approach was found efficient due to a joint approach in the learning process. Trisedya et al.^22^ studied knowledge graphs and attribute embeddings to achieve entity alignment. Hosseinzadeh et al.^23^ studied various methods and application mechanisms to clean data towards big data analytics. Pita et al.^24^ proposed a system for probabilistic data linkage and identification of duplicates with high scalability and accuracy. Li et al.^25^ investigated on data management in the context of crowdsourced data available while EnAli is the framework proposed in^26^ for alignment and consistency of entities. Table 1 shows a summary of important findings in the literature concerning deduplication of entities.

**Table 1:** Shows important findings from the literature.

Reference	Method	Embedding Model	Alignment Model	Extra information	Prediction	CL	ML
^22^	AttrE	TransE	A	Attribute	CS	NO	Yes
^60^	BootEA	TransE variant	C	Bootstrapping	CS	Yes	Yes
^61^	CEA	GCN	M	Entity name	CS	Yes	Yes
^62^	GCN-Align	GCN	M	Attribute	MD	YES	NO
^63^	GM-Align	GCN	G	Entity name	MP	Yes	No
^21^	HGCN	GCN	M	Entity name	MD	Yes	No
^64^	HUMAN	GCN	M	Description, Attribute	ED	Yes	No
^16^	ITransE	TransE	T	Bootstrapping	ED	No	Yes
^65^	JAPE	TransE variant	A	Attribute	CS	Yes	No
^66^	KDCoE	TransE	T	Entity description	ED	Yes	No
^15^	KECG	GAT + TransE	M	No	ED	Yes	Yes
^13^	MTransE	TransE variant	T	No	ED	Yes	No
^10^	MuGNN	GNN	M	No	CS	Yes	Yes
^67^	MultiKE	TransE variant	C	Entity name	CS	No	Yes
^68^	NAEA	TransE variant	C	Bootstrapping	CS	Yes	Yes
^69^	RDGCN	DPGCNN	M	Entity name	MD	Yes	No
^14^	RSNs	RSNs	C	No	CS	Yes	Yes
^70^	TransEdge	TransEdge	C	Bootstrapping	CS	Yes	Yes

(C-corpus function, M-margin based, A-attribute refined, G-graph based, T-transition, ED-Euclidian distance, CS-cosine similarity, MD-Manhattan distance, MP-matching probability, CL for cross-lingual and ML for mono-lingual).

Xia et al.^27^ studied existing data deduplication methods. Their research threw light on different aspects of entity resolution or deduplication covering heuristics and learning-based methods. Aalberg et al.^28^ studied bibliographic records and various available methods for their deduplication and benchmarking of them in terms of evaluation and interpretation. Zheng et al.^11^ proposed a tool known as SEM + which is used to eliminate data inconsistencies and discover concept mapping in various domains like earth science. Hosseinzadeh et al.^29^ focused on various data-cleaning mechanisms that are used to improve the quality of big data. Hörsch et al.^30^ proposed a model known as PyPSA-Eur which is designed for optimising data and improving a data transmission system. Saberi et al.^31^ considered databases of various organizations to deal with quality management methods for the elimination of duplicate records in the data. Table 2 shows a summary of different techniques, their merits and limitations.

**Table 2:** Techniques used in prior works along with their merits and demerits.

Reference	Technique	Dataset	Advantages	Limitations
^71^	ML-based methods	Real-world datasets	High accuracy, scalability and efficiency	Usability is low
^10^	A statistical approach to treat missing values	Custom datasets	High accuracy, scalability and efficiency	Usability is low
^9^	ML for generation of match classifiers	Custom dataset	High scalability and efficiency	Usability and accuracy are low
^12^	Ensemble with noise elimination	Smart Dataset	High scalability and high efficiency	Usability and accuracy are low and complexity is high
^8^	Bayesian generative process model	Car sales dataset	High scalability, accuracy and efficiency	High complexity and usability are low
^72^	Classification rules configuration	RDD	High accuracy and efficiency	Usability and scalability are low

Kawka et al.^4^ proposed an ML models-based system for automatic analysis of video content towards ascertaining the content and also eliminate inconsistencies. Different approaches to entity resolution are explained in^32^ while ML models are used in^5^ for automatic processes towards data quality improvement. Negahban et al.^33^ used deep learning along with transfer learning to expedite the learning process and perform entity resolution based on knowledge gained. Gabriel et al.^34^ considered the healthcare domain particularly clinical data to identify similar entities and thus avoid duplicates towards improving the quality of data for diagnosis. Papadakis et al.^35^ proposed an efficient entity resolution methodology considering information spaces coming from diversified sources. Jia et al.^36^ focused on video deduplication methodology by proposing a method based on aggregation of triplet loss feature and scalable hash. Lee^37^ proposed a computer-aided system to deal with archival material and improve the quality of data with different techniques.

Xinyao et al.^38^ suggested method provides excellent user-defined access control and secure deduplication, protecting data confidentiality and successfully fending off threats. Elouataoui et al.^39^ discussed safe deduplication techniques for cloud storage that boosts storage effectiveness while protecting data confidentiality and integrity. Cho et al.^40^ Storage optimization solutions—which fall into three categories: content-based, redaction, and replication—are required due to the increase in blockchain transactions. Challenges with these systems include security, decentralization, and scalability. Zhou et al.^41^ by combining comparable data bits, granulation of information improves machine learning performance and facilitates feature selection for Big Data jobs. Alluhaidan et al.^42^ suggested a duplicated storage adaptive migration technique that maximizes space savings and service flexibility. It reduces the cost of additional space and enhances data availability by utilizing heuristics and ILP. Borissov et al.^43^ entered data integration are resolved using entity resolution, or ER. By reducing thorough pairwise comparisons, blocking increases productivity. The efficacy and efficiency of the rough set approach are improved. Shen et al.^44^ suggested safe encrypted deduplication method for cloud storage ensures efficiency and security by enabling deduplication without the help of a third party. Jiang et al.^45^ improved user happiness and interaction success rate, the Cloud Services Trust Evaluation Model (CSTEM) integrates weights and grey correlation analysis. Through simulation trials, it combines reputation, recommended trust, and direct trust to provide a thorough trust evaluation. Menaouer et al.^46^ for JointCloud storage, the Secure and Efficient data Deduplication (SED) strategy is suggested, which improves security, functionality, and efficiency. Menaouer et al.^47^ increased precision without compromising recall, a hybrid recommendation system for healthcare makes use of rough set pruning. Koumarelas et al.^48^ proposed a duplicate detection method named MDedup which is a rule rule-based approach with a matching dependencies concept. A hierarchical NN-based model is proposed in^49^ for duplicate entity detection using a deep string matching approach. Both^48^ and^49^ are used in this paper for comparison with the proposed model. Zhao et al.^50^ explored entity alignment methods to ascertain their modus operand and empirical findings, while Vatsalan et al.^51^ focused on challenges in record linkage and privacy-preserving issues for big data. From the literature, it is understood that deduplication research of late is considering machine learning and deep learning-based approaches for efficiency and scalability. As we explored in^6^, there is a deep learning-based model for entity resolution with certain variants. This model was found to be effective and very useful towards data deduplication. However, we found that it could be improved with many extensions such as numeric attribute support, null values support and trainable embeddings that were missing in the existing model.

## Preliminaries

This section presents preliminary information required to ascertain the work in this paper. It throws light on various aspects such as word embeddings, learning-based approaches and existing deep learning-based methods.

### Word embeddings

Word embeddings is the phenomenon which maps given tokens into a vector space in such a way that similar tokens even in terms of semantic meanings can be grouped as discussed in^52^. Stated differently, tokens like PC and computer reflect high cosine similarity. To create embeddings there are many methods such as a bag of words, skip-gram and matrix factorization^53^ to mention a few. All different kinds of embeddings share an assumption that the meaning of a word depends on its context in association with neighbouring words. For instance, words such as congress and senate are words closer to words like politics and government. Considering a text corpus with size v in terms of vocabulary, it is possible to naively embed words in the form of a v-dimensional vector the elements provide the frequency of the word in the neighbourhood of w in the given corpus. However, such a naïve method is generally impractical as v could be very large.

**Figure 1:**
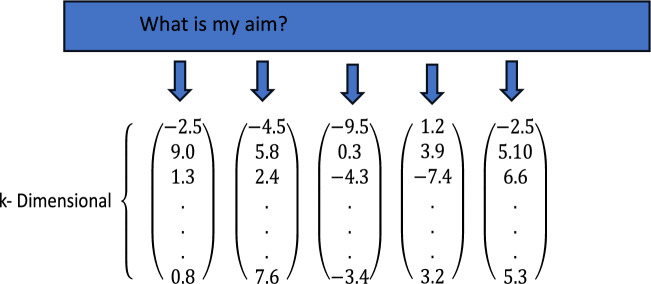
Illustrates the idea of word embeddings.

GloVe explored in^53^ is an algorithm for word embeddings. It is based on the matrix factorization approach. Provided a set of documents in the corpus, it is possible to generate a covariance matrix, denoted as $X \in {\mathbb{R}}^{\upsilon \times \upsilon }$. Here ${w}_{j}$ denotes a word and ${X}_{ij}$ indicates the number of times the word is found in a given set of words in a corpus. GloVe performs matrix factorization resulting $W\in {\mathbb{R}}^{\upsilon \times k}$. W denotes the entire matrix as the embedding layer associated with a neural network. In the baseline reference model^6^, 300-dimensional pre-trained word embeddings that are generated from a corpus containing 840 billion tokens. Figure 1 illustrates the idea of word embeddings.

### Learning-based approaches

**Figure 2:**
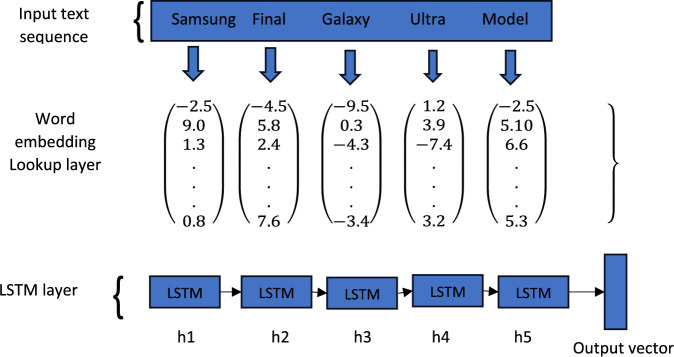
Illustrates usage of LSTM.

Learning-based approaches are based on machine learning and they learn from given training data either implicitly or explicitly. Neural networks and deep learning modes are found to be efficient learning-based approaches with a high degree of accuracy. Neural network models take input vectors and perform their operations such as matrix multiplications to generate output vectors as explored in^7^. Such networks are trained with the help of gradient descent. Then output error is utilized to compute the error gradient that helps in taking steps to minimize error. About NLP, the first layer of the network acts as an embedding layer that takes one hot vector as input and performs appropriate embedding. It is also possible to fix the weights of the embedding layer towards gradient-based updates given to subsequent layers to make the weights of the layer trainable^1^. LSTM is a variant of CNN which could model sequential dependencies linked to given data. Considering ${x}_{1}$, ${x}_{2}$, …, ${x}_{T}$ as a sequence of input vectors, RNN needs the previous step's output. Thus, the output of RNN consists of all prior vectors in the time steps^54^. RNN has an important drawback in modelling long-term dependencies. To overcome this issue, LSTM came into existence^55^ by adding memory cells to RNN. The usage of LSTM is illustrated in Fig. 2.

Such memory cells carry information through different time steps solving long-term dependency problems. Therefore, LSTM models achieved success in various domains like sentiment analysis, text classification and machine translation. An extension to LSTM is known as bi-LSTM where another LSTM is used for the same data in different directions to have another view of data^56^. The final output of bi-LSTM is the concatenation of the result of two LSTMs.

### MaLSTM

**Figure 3:**
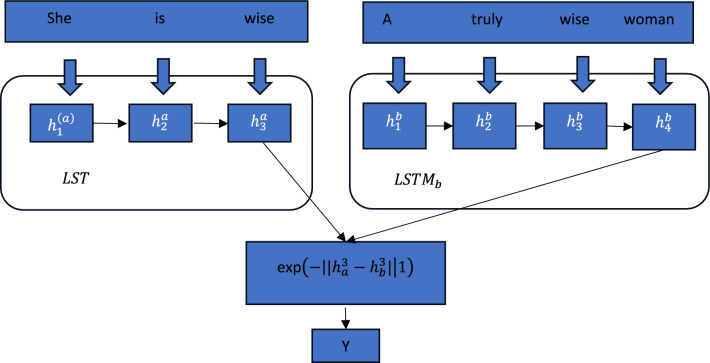
Illustrates the usage of LSTM with Manhattan Metric.

Typically, neural networks need an object as input in the form of an image, tuple or document. Sometimes, pairs of objects are involved in distance computations towards generating desired output^9^. MaLSTM is a variant of the LSTM model where the Manhattan distance metric is used. Siamese network is a common neural network that supports distance metric learning. Figure 3 shows the architecture of MaLSTM.

As discussed in^57^ MaLSTM is based on Siamese architecture for scoring sentence similarities. The outcome of two LSTMs denoted as $x \epsilon {\mathbb{R}}^{d}$ d and y $\epsilon {\mathbb{R}}^{d}$ are combined through similarity function on top of $s\left(x,y\right)$ = ${e}^{{-\Vert x-y\Vert }_{1}}$ which is known as l1-based similarity. By using such a similarity function LSTMs involved in the architecture can find semantic differences between two sentences. MaLSTM is known for its efficiency in finding sentence similarities. However, in this paper, we explored it to know its performance for record tuples that are text-based.

## Reference model

In this paper, we used DeepER^6^ as a reference model. This model is similar to that of MaLSTM in terms of using two LSTM networks. However, the outcomes of the two networks are not directly combined. Instead, the outcomes are transformed into a distributed similarity vector. Section "Proposed algorithm" presents more details about the reference model.

## Methodology

This section presents our proposed framework and its modus operandi in providing suitable extensions to the method in^6^ to address its limitations.

### Problem definition

The existing deep learning-based method in^6^ for the elimination of duplicates in data is found efficient. However, its potential has not yet been examined under varying embeddings and text processing methods. Another important limitation is that the existing method could handle only textual attributes. However, in reality, there are data sources with numeric attributes as well. Another observation is that there is a need for dealing with missing values as well. Support for many similarity metrics and varied methodologies for word embeddings is yet to be investigated. In this paper, we make the required extensions to overcome these limitations.

#### Baseline framework

**Figure 4:**
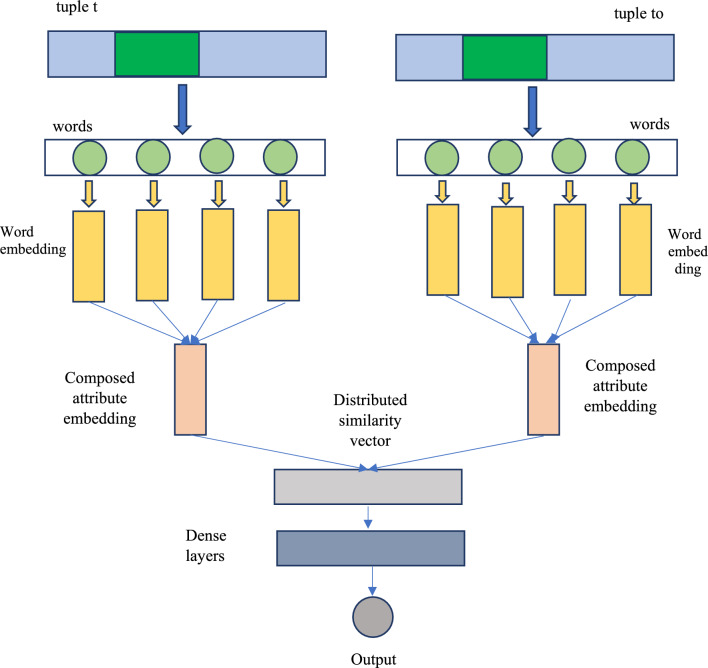
The baseline framework for learning-based deduplication.

To investigate on the limitations mentioned in Section "Problem definition", we proposed extensions to the framework of^6^ shown in Fig. 1. The framework is based on a deep learning model which helps in learning from training data before performing duplicate record identification. Duplicate record identification is a classification task that distinguishes duplicate records. The baseline architecture is very much similar to that of LSTM with the Manhattan measure. The Manhattan measure-based LSTM exploits two similar architectures with underlying weights. However, it is to be noted that the outcome of the two architectures is not explicitly mapped to a measure of similarity. Instead, the two outcomes are clubbed to form a similarity vector which is distributed in nature. This approach is in tune with many ML-based methods used for record deduplication. The LSTM with Manhattan measure does have hidden layers that are densely connected. The outcomes are mapped to a binary vector reflecting the discrimination capability of the model as discussed in^6^. A tuple can have many attributes and each one can have several tokens. Replacing such tokens with corresponding word embedding leads to a matrix. In such a case, it is not easier to compute inter-attribute similarity. The baseline framework shown in Fig. 4, however, composes attributes into vector form for ease of processing.

The framework supports two kinds of composing. A single k-dimensional vector is generated from different word embedding vectors using the average method. Then LSTM is applied to the resultant vector to generate a new vector. This approach is known as the shared LSTM method. In this approach, a single LSTM is used for generating attribute embeddings. With the averaging method, the baseline approach uses cosine similarity which maps 2 k-dimensional vectors to a single scalar value. Thus the resultant scalar values are concatenated to have a similarity matrix which is distributed in nature. As mentioned earlier another method used by the baseline model for generating embeddings is LSTM.

When LSTM-based composition is applied, it results in k'-dimensional and the final result is a vector with concatenated embeddings. Finally, the dense layers present in the baseline framework are responsible for mapping vectors into binary values 1 (indicating duplicate) and 0 (no duplicate). Notations used in this paper are provided in Table 3.

**Table 3:** Notations used in this paper.

Notation	Description
${A}_{j}$	Denotes jth attribute
${}_{VL}$	Denotes word embedding of ${w}_{l}$
A	Denotes an attribute
a, b	Indicate two scalar values
D	Denotes dataset
df(w)	Indicates count of tuples where token w is found among attributes
idf(w)	Denotes inverse document frequency
N	Represents pairs of tuples
S	Denotes a vector
T	Denotes a tuple
x, y	Denote two k-dimensional vectors
Y	Denotes a label vector
α	Denotes smoothing hyperparameter

#### Proposed extensions to reference model

This section presents our proposed extensions made for the reference model. The extensions make the model to leverages its functionality.

#### Adaption of LSTM with Manhattan measure

The model is enhanced with the adaption of LSTM with Manhattan measure for deduplication. This could help in simple adjustments to data. Attributes of data are tokenized for each tuple and then each token is converted to word embedding. Afterwards, all embeddings are concatenated. Here lies the intuition that this LSTM variant is better in detecting similarities and its effectiveness is not impacted even when attribute demarcations are removed in a given tuple to make it a sentence. This variant along with learning distance metrics could improve performance but not significantly. Further investigations are given in the subsequent sections.

#### Attribute compositions

##### Inverse document frequency

As presented in the reference model^6^, it could perform well when LSTM is used for attribute embeddings instead of a simple averaging method. However, it is also observed that the averaging method could provide significantly better results in some cases. Moreover, it is observed that the averaging method is less expensive in resources and time when compared with that of LSTM. Nevertheless, there is one weakness with the averaging method which is the treatment of all tokens equally while in reality, some tokens might be more informative. In NLP there is a common practice to adjust weights of tokens based on their occurrence. This is achieved by computing TF-IDF as discussed in^15^. In^6^ word embeddings are done towards composing attribute embeddings by considering token frequency associated with attributes. When an attribute has the presence of a token two times, averaging is done twice to achieve the attribute embedding process. For this reason, down-weighting of tokens is our focus based on their presence in several tuples. For a given token, denoted as w, its inverse document frequency is computed as in Eq. (1).

1 $$idf\left(w\right)=\frac{1}{{df\left(w\right)}^{\frac{1}{\propto }}}$$

where the count of tuples containing the presence of w among attributes is denoted as df(w) and α denotes a smoothing hyperparameter which takes care of balance in down weighting process. For given attribute A, its attribute embedding process is expressed in Eq. (2).

2 $$\upsilon =\frac{{\sum }_{l=1}^{L} idf\left({w}_{1}\right){\upsilon }_{1}}{{\sum }_{l=1}^{L} idf\left({w}_{1}\right)}$$

where ${w}_{1}$∈ A indicates a token associated with attribute A, ${\upsilon }_{l}$ denotes word embedding related to ${w}_{l}$ and v refers to the attribute embedding linked to A. For a given attribute IDF is computed for every token and then weights are normalized to get a sum of 1. Thus word embeddings' weighted average is the actual embedding for a given attribute where coefficients are used as inverse document frequencies.

##### Enhanced compositions

As mentioned earlier, attribute-related computations are based on datasets. With the help of averaging process-based composition attribute embeddings may do better when compared with LSTM composition and vice versa as well^6^. And there are experiments with reference models to know which composition is optimal. Therefore, a natural step forward with the reference model is to automate the choosing of one of the two compositions to improve performance in the deduplication process. Improvement made in this paper is that, for a given attribute pair, generation of averaging and LSTM-based embeddings and achieving a compound composition where similarity measures are computed for each attribute pair. Further extension of this could lead to IDF-based embeddings resulting in 3 measures for similarity for a given attribute pair.

#### Usage of Multiple Similarity Metrics

As multiple methods are available for attribute embedding in the reference model, it is possible to extend it to support multiple similarity metrics to be used with each pair of embedding attributes. In the original reference model, as explained in^6^, the averaging method composition is evaluated with the help of cosine similarity while LSTM composition is transformed into difference and concatenated to form a distributed similarity vector. As cosine similarity is found good, it may be used in LSTM composition also as an additional metric. Further, the reference model is extended to exploit the ${l}_{1}$-based similarity measure which is being used in LSTM with Manhattan measure. The similarity measure for x and y, the two k-dimensional vectors, is as expressed in Eq. (3).

3 $$s\left(x,y\right)={e}^{{-\Vert x-y\Vert }_{1}}$$

This measure is useful for any kind of composition. Our original measure used for Gaussian kernels computation is as in Eq. (4).

4 $$s\left(x,y\right)={e}^{{-\Vert x-y\Vert }_{2}^{2}}$$

Nevertheless, prior studies such as^58^ reported the fact that it leads to vanishing gradients resulting in untrainable networks. With empirical study, we came to know that distributed similarity vector involves the usage of l2 similarity. Moreover, we consider the conversion of average compositions into difference and product vectors. However, we turned against this idea due to the dimensionality issue.

#### Support numerical attributes

The reference model in^6^ did not support numerical attributes without converting them into categorical attributes. This approach was not optimal due to the difference in semantic differences of values when converted to strings. To support numerical attributes, a similarity measure is used to compare pairs of scalar attributes of numeric type. Towards this end, we proposed measures for two scalars named a and b as expressed in Eqs. (5, 6 and 7).

5 $$s\left(a,b\right) = {e}^{-\left|a-b\right|}$$

This is known as an unscaled Gaussian measure while Eq. (6) is known as a scaled Gaussian measure.

6 $$s\left(a,b\right)= {e}^{- \frac{2\left|a-b\right|}{a+b}}$$

Another measure known as the min–max ratio is given in Eq. (7).

7 $$s\left(a,b\right)=\frac{min\left(a,b\right)}{max\left(a,b\right)}$$

These similarity measures are meant for numerical attributes. However, they expect non-negative values in the attributes. If zero is the value for both a and b, the min–max ratio and scaled Gaussian measures are technically undefined. In such case 1 is assigned as similarity to the scalar pair. These measures can be computed collectively and concatenated to have a distributed similarity vector.

#### Support for null value indicators

Null values may occur in both categorical and numerical attributes. In either case, imputing is essential. In the case of numerical attributes, assigning 0 to missing values and in the case of categorical attributes assigning "NaN" as a placeholder token can be done. However, this approach throws challenges in the reference model^6^. To overcome this problem, if the two attributes in a pair have nulls, imputing them with the same value that results in similarity 1 is done or ensuring that the product vector of both is the same. This has the potential to dilute information associated. To address this problem, we introduce a binary indicator attribute for each categorical and numerical attribute. The binary indicator value is 1 if there is a null value otherwise 0. Thus, under this process, a pair of tuples with 2 attributes is transformed into a pair of tuples with 4 attributes. Then while computing the distributed similarity vector, all null value replacements are considered. They are concatenated directly without using any intermediate measure.

### Proposed algorithm

We proposed an algorithm named Efficient Learning based Record Deduplication (ELbRD) which reflects the enhancements to the reference model.

**Algorithm 1:**
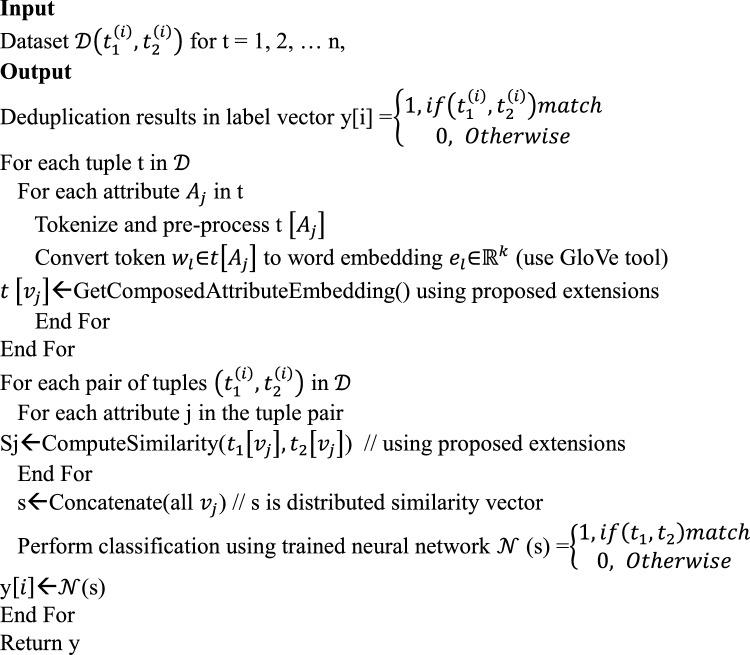
Efficient Learning-based Record Deduplication (ELbRD).

As presented in Algorithm 1, it takes dataset $\mathcal{D}\left({t}_{1}^{\left(i\right)},{t}_{2}^{\left(i\right)}\right)$ for t = 1, 2, … n to be used for deduplication as input and produces results in the form of a vector reflecting duplicate records for elimination. The algorithm starts with a nested iterative process where each tuple in a given dataset and each attribute in the tuple are processed. The processing involves pre-processing, tokenization, converting tokens ${w}_{l}$∈$t\left[{A}_{j}\right]$ into word embeddings ${e}_{l}$∈${\mathbb{R}}^{k}$ (using the GloVe tool) and finally obtaining composite attribute embeddings based on the enhancements proposed to the baseline model. Afterwards, there is another nested iterative process where each pair of tuples $\left({t}_{1}^{\left(i\right)},{t}_{2}^{\left(i\right)}\right)$ in and each attribute underlying are processed. This processing includes similarity computations based on the proposed extensions, generation of distributed similarity vectors and classification of the tuples to discriminate duplicates in the dataset.

## Experimental setup

This section provides details related to datasets, steps involved in pre-processing, and evaluation procedure to know the efficiency of extensions to reference model in^6^. Three datasets are used in the empirical study.

### Datasets

Benchmark datasets used in^6^ and^59^ are used for experiments in this paper. The data is structured in nature. The underlying data in each dataset comes from various sources. The three datasets details are given in Table 4.

**Table 4:** Dataset details.

Dataset	#Tuples from amazon	#Tuples from google	#Matches	#Attributes
Amazon-google	1363	226	1300	5
Amazon-Walmart	2554	22,074	1154	17
DBLP-scholar	2616	64,263	5347	4

The datasets used in the experiments contain data in the form of a tabular format consisting of several attributes and tuples. Each dataset is ensured to have a certain number of matches or duplicates.

### Pre-processing

The pre-processing involves tokenization of categorical attributes to transform them into word embeddings. Unlike the reference model^6^, we perform pre-processing for both numerical and also text attributes. In the process, we used two schemes for tokenization. They are known as the standard approach and the full approach. In the former, tokens are transformed into lowercase while punctuations are removed. Single-character tokens are also removed. Tokens that appear 10% or higher in tuples are also removed. Thus, it could reduce the number of unique tokens present in the dataset. However, it has a problem with losing semantic distinctions between words while converting them into word embeddings. In the case of the full approach, it makes use of all unique tokens without discarding them. This approach preserves the original text as it minimizes alternations. It also preserves semantic meaning in the text but it may lead to an overfitting problem. GloVe tool is used to convert tokens into word embeddings that have been trained using the corpus in^53^ which has 2.2 million words. If there is a token that is not in the trained vocabulary, it is mapped to 0's vector instead of ignoring it. Thus, unknown tokens are mapped to vectors and this helps neural networks trainable. However, care is taken to prevent adding noise while performing average-based compositions. In the reference model^6^, no information is given on handling missing data. In our work, we followed the imputation process explained earlier.

### Validation and environment

Each dataset, as per prior benchmark tests, satisfies the 1:100 match and non-match ratio. According to this, it can be understood that the number of non-matches is very high. Therefore, to address this problem, we incorporated negative sampling to bring balance to the datasets. Once it is completed, we split data into training sets (80%), validation sets (10%) and test sets. Unlike the work in^6^, we do not follow blocking in the validation scheme. With this decision, our proposed extensions to the baseline model could perform better in terms of F1-Score.

The environment used for experiments in this paper is provided in Table 5. Experiments are made with different variants of LSTM and many extensions are made to reference model.

**Table 5:** Environment used in empirical study.

Processor	Intel Xeon 8-core E5-2623
GPGPU	NVIDIA P5000
RAM	16 GB

## Results and discussion

We built a prototype to evaluate the proposed learning-based method for record deduplication. This section presents experimental results. Different models used for empirical study are briefly described here. Avg is the model that is baseline in^6^ which follows standard tokenization, averaging composition, and cosine similarity measure and does not support trainable embedding, null values and numeric attributes. Lstm is another baseline approach in^6^ with standard tokenization, bi-lstm for composition, difference and Hadamard similarity measures, and no support for trainable embedding, null values and numeric attributes. Avg-t is another baseline method which uses standard tokenization, averaging composition, and cosine similarity measure and does not support null values and numeric attributes. However, it supports trainable embedding. Lstm-t is yet another baseline method which uses standard tokenization, bi-lstm composition, difference and Hadamard similarity measures and does not support null values and numeric attributes. However, it supports trainable embedding. Apart from these baseline methods, many methods are improved on top of reference models in^6^ as discussed below.

Idf is a model which uses standard tokenization, composition includes averaging, idf and bi-lstm, cosine similarity and does not support null values, numeric attributes and trainable embedding. Allcomp is a model which uses standard tokenization, composition includes averaging, idf and bi-lstm, similarity includes cosine, difference and Hadamard and does not support null values, numeric attributes and trainable embedding. Avg-allsim is a model which uses standard tokenization, average composition, and similarity including cosine and l1 and does not support null values, numeric attributes and trainable embedding. Lstm-allsim is a model which uses standard tokenization, bi-lstm composition, similarity includes cosine, difference, Hadamard and l1 and does not support null values, numeric attributes and trainable embedding. Allcomp-all sim is a model which uses standard tokenization, composition includes idf, bi-lstm and averaging, similarity includes cosine, difference, Hadamard and l1 and does not support null values, numeric attributes and trainable embedding. Allcomp-all sim-t is a model which uses standard tokenization, composition includes idf, bi-lstm and averaging, similarity includes cosine, difference, Hadamard and l1 and does not support null values and numeric attributes. However, it supports trainable embedding.

Avg-full is a model which uses standard tokenization, composition includes idf, bi-lstm and averaging, similarity includes cosine, difference, Hadamard and l1 and does not support null values and numeric attributes. However, it supports trainable embedding. Lstm-full is a model which uses full tokenization, bi-lstm composition, similarity includes difference and Hadamard and does not support null values, numeric attributes and trainable embedding. Avg-num is a model which uses standard tokenization, averaging composition, and cosine similarity and does not support null values and trainable embedding. However, it supports numeric attributes. Avg-num-null is a model which uses standard tokenization, averaging composition, and cosine similarity and does not support trainable embedding. However, it supports numeric attributes and null values. Avg-num-t is a model which uses standard tokenization, averaging composition, and cosine similarity and does not support null values. However, it supports numeric attributes and trainable embedding. Avg-num-null-t is a model which uses standard tokenization, averaging composition, and cosine similarity and supports null values, numeric attributes and trainable embedding. Avg-allsim-num-null-t is a model which uses standard tokenization, averaging composition, cosine and l1 similarity and supports null values, numeric attributes and trainable embedding. Lstm-num is a model which uses standard tokenization, bi-lstm composition, difference and Hadamard similarity and supports numeric attributes. However, it does not support null values and trainable embedding. Lstm-num-null is a model which uses standard tokenization, bi-lstm composition, difference and Hadamard similarity and supports numeric attributes and null values. However, it does not support trainable embedding. Allcomp-allsim-num-null-t is a model which uses standard tokenization, composition includes difference, l1, Hadamard and cosine and supports numeric attributes, null values and trainable embedding.

As presented in Table 6, the performance of LSTM variants and the average method are provided in terms of the F1 score.

**Table 6:** Performance of LSTM variants and Avg.

Dataset	F1-score			
	avg	Lstm	Malstm	Malstm-t
Amazon-google	0.65	0.81	0.612	0.6
Amazon-Walmart	0.84	0.85	0.72	0.75
dblp-scholar	0.96	0.97	0.85	0.85

**Figure 5:**
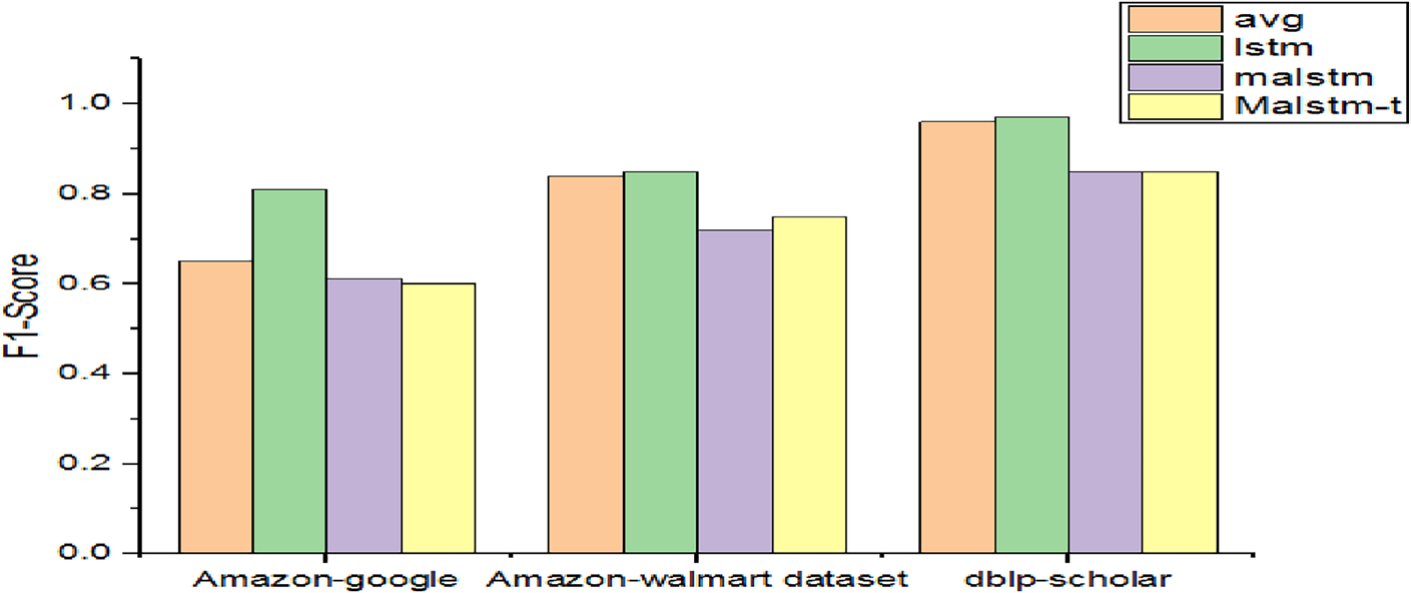
Performance comparison among LSTM variants and Avg.

Malstm is evaluated with trainable embedding while Malstm-t is evaluated with un-trainable embedding. It is observed with experiments that malstm and malstm-t are not impressive for record deduplication tasks as shown in Fig. 5. As discussed in^57^ maelstrom has issues with short sentences and training with long sentences. The highest F1-Score is exhibited by the Lstm model for all datasets when compared with other models. Its highest F1-Score is reported, when dblp-scholar data is used, with 97%.

As presented in Table 7, the performance of various methods is compared in terms of F1-Score. Out of them Avg and Lstm are baseline models.

**Table 7:** Performance with different attribute embedding methods.

Dataset	F1-score			
	Avg	Idf	Astm	all comp
Amazon-google	0.66	0.73	0.81	0.84
Amazon-Walmart dataset	0.84	0.812	0.855	0.855
dblp-scholar	0.97	0.955	0.975	0.98

**Figure 6:**
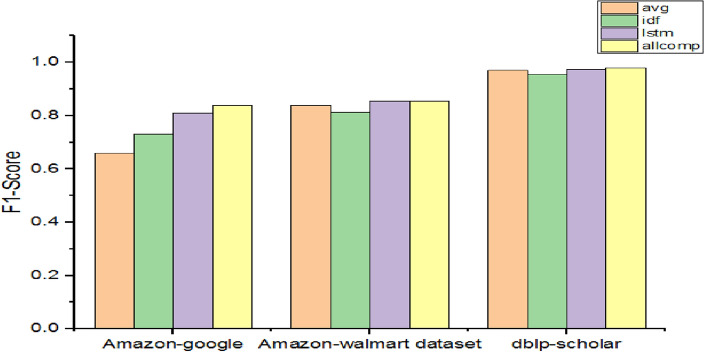
Performance comparison among various attribute embedding methods.

As presented in Fig. 6, there are benefits of using IDF weightings for embeddings in the case of the Amazon-Google dataset. However, with the other two datasets, idf showed less performance when compared with the Avg method. The based approach for attribute embedding showed relatively better performance over idf and Avg. The highest F1-Score among the three datasets exhibited by the Avg method is 97%, idf 95.5%, lstm 97.5% and allcomp method 98%. The highest among the methods is achieved by allcomp with 98% when the dblp-scholar dataset is used.

Table 8 presents the performance of different models used in experiments with different similarity measures. Observations are made on three datasets in terms of F1-Score.

**Table 8:** Performance with different similarities.

Dataset	F1-score					
	avg	Avg-allsim	lstm	Lstm- allsim	Allcomp- allsim	Allcomp- allsim-t
Amazon-google	0.66	0.71	0.81	0.7725	0.78	0.80
Amazon-Walmart dataset	0.84	0.83	0.86	0.869	0.87	0.869
dblp-scholar	0.96	0.965	0.967	0.969	0.97	0.975

**Figure 7:**
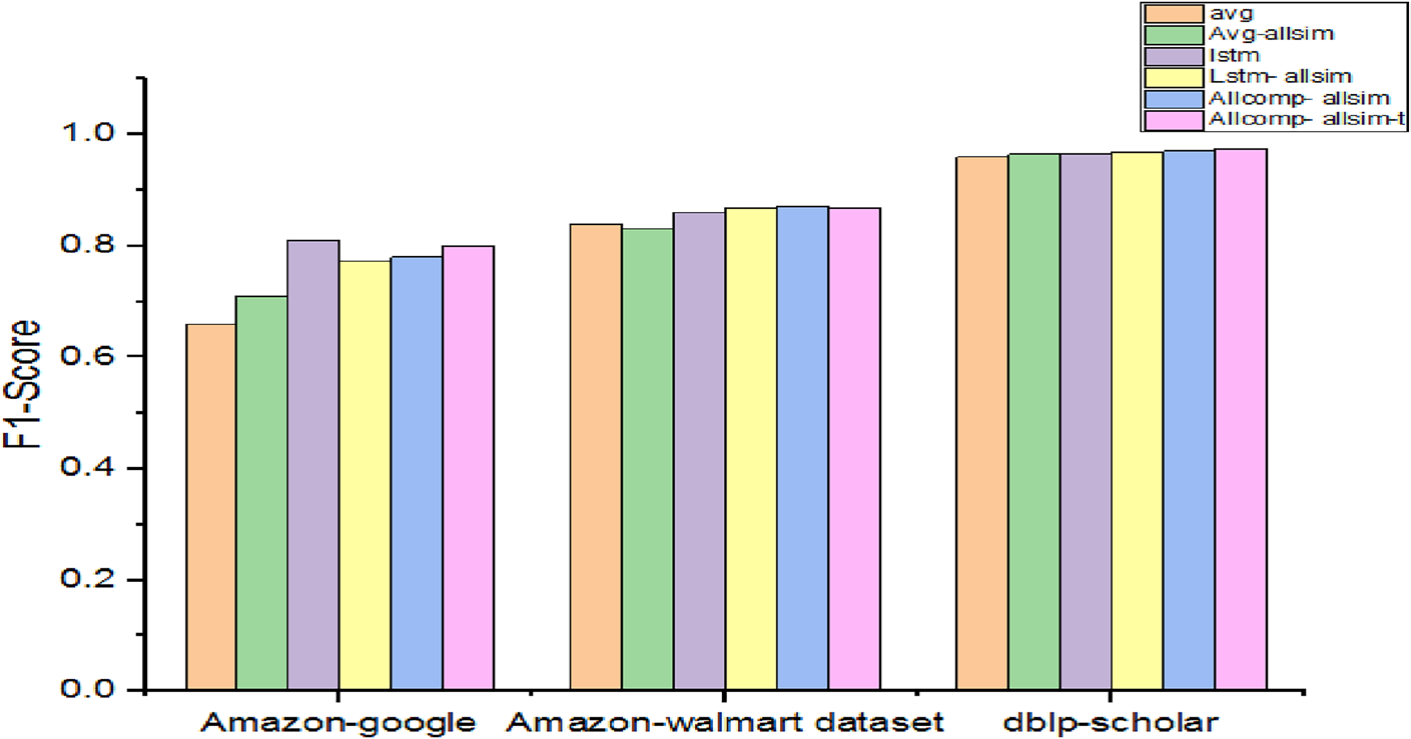
Performance comparison among various attribute embedding methods.

As presented in Fig. 7, the method showed the highest performance using the dblp-scholar dataset with 96% F1-Score. However, this performance is least when compared with other models for the same dataset. Allcomp-all sim-t mode showed highest performance with 97.5% F1-Score for the dblp-scholar dataset. There is notable performance improvement visible when multiple embeddings are used. However, multiple similarities in usage in this case did not show much difference in performance.

As presented in Table 9, the baseline models without the proposed extensions are evaluated for their performance.

**Table 9:** Performance with different baseline methods associated with a reference model.

Dataset	F1-score			
	avg	Avg-t	lstm	Lstm-t
Amazon-google	0.66	0.86	0.81	0.823
Amazon-Walmart	0.84	0.875	0.855	0.82
dblp-scholar	0.96	0.965	0.975	0.98

**Figure 8:**
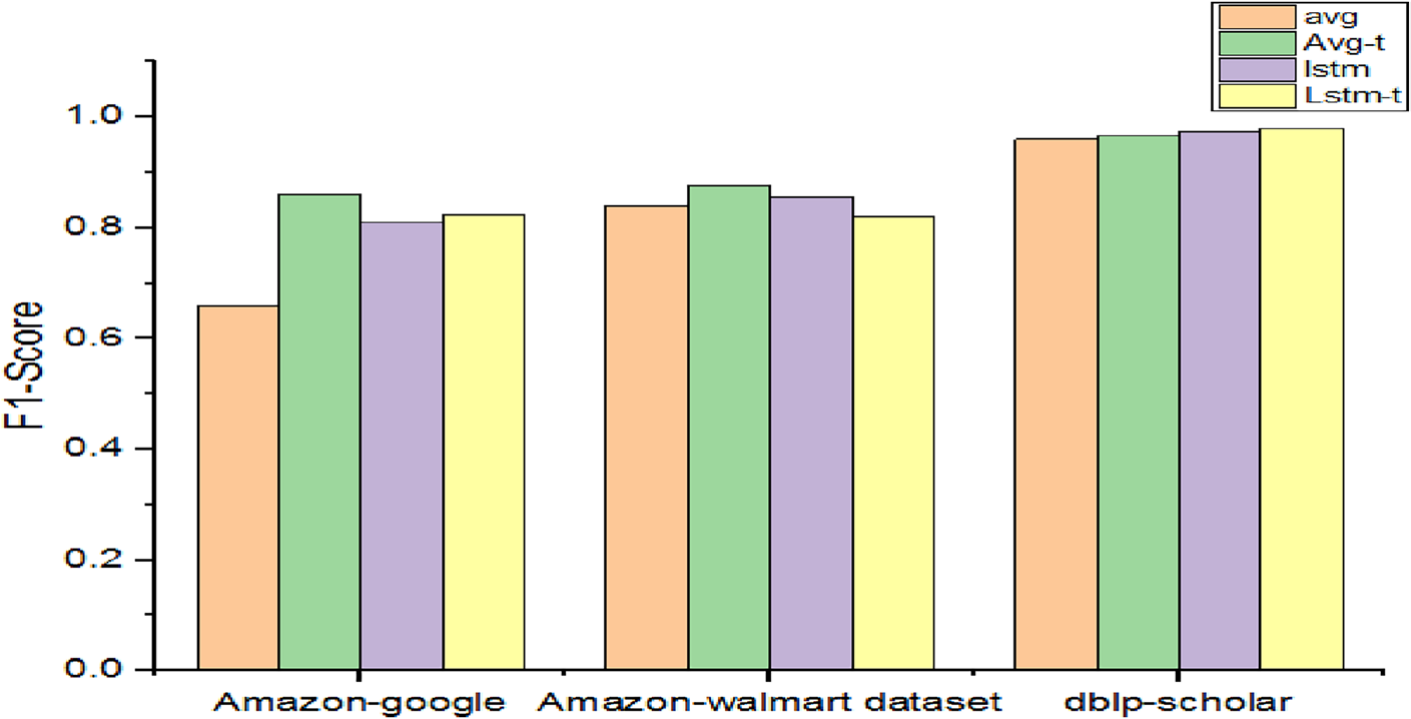
Performance comparison among variants of DeepER.

As presented in Fig. 8, the results of baseline models are provided. The highest F1-Score is exhibited by lstm-t when the DB-scholar dataset is used as it makes use of difference and Hadamard as similarity measures along with trainable embedding and bi-lstm-based composition. Lstm also uses similar kinds of configurations as that of bi-lstm but it does not support trainable embedding. Therefore, its performance is less than last with a 97.50% F1-Score. Avg-t showed better performance over avg due to trainable embedding support. It is observed that trainable word embeddings have an impact on the performance of the models.

As presented in Table 10, models are observed in terms of their performance when two kinds of pre-processing are employed.

**Table 10:** Performance with improved pre-processing method.

Dataset	F1-score			
	avg	Avg-full	Lstm	Lstm-full
Amazon-google	0.66	0.649	0.81	0.79
Amazon-Walmart	0.84	0.82	0.86	0.84
dblp-scholar	0.97	0.89	0.975	0.98

**Figure 9:**
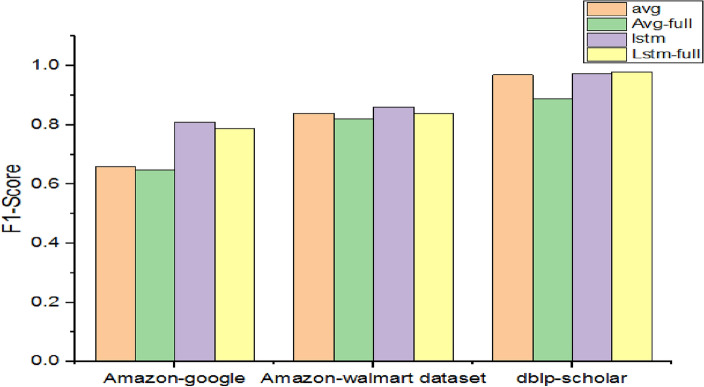
Performance comparison among methods with improved pre-processing.

As presented in Fig. 9, there is a visible difference between full and standard tokenization methods in pre-processing. Since full tokenization enjoys complete vocabulary, has its limitations in terms of performance. Though full tokenization enhances semantic distinctions, it suffers in performance degradation due to overfitting caused by larger vocabulary.

As presented in Table 11, experiments made with models consisting of varying support for numeric attributes and null values showed their impact on performance.

**Table 11:** Performance with different numerical attribute embeddings.

Dataset	F1-score				
	avg	Avg-t	Avg-num-t	Avg-num-null-t	Avg-num-null-allsim-t
Amazon-google	0.66	0.86	0.88	0.98	0.8
Amazon-Walmart	0.84	0.87	0.91	0.875	0.87
dblp-scholar	0.96	0.965	0.97	0.97	0.956

**Figure 10:**
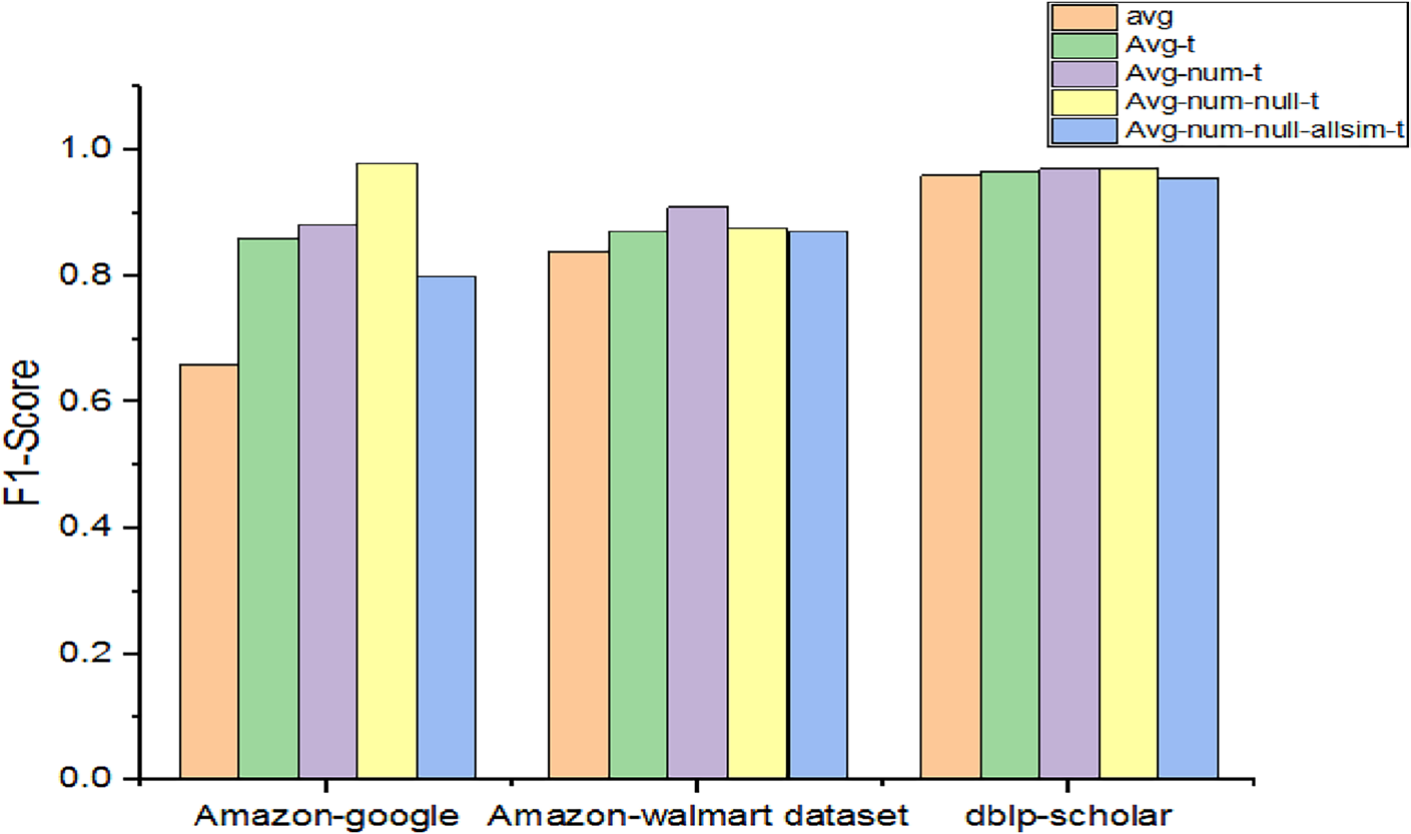
Performance comparison among methods with different numerical attribute embeddings.

As presented in Fig. 10, incorporating support for numeric attributes and null values made a difference in performance in the discrimination of duplicates. Since avg-t supports trainable embedding, its performance is better than the average model. However, other avg variants showed better performance than avg and avg-t due to the incorporation of numeric attributes and null values. The highest performance is achieved by Avg-num-t and Avg-num-null-t using the dblp-scholar dataset with 97% F1-Score.

As presented in Table 12, the performance of four baseline models is compared with the model with all proposed extensions.

**Table 12:** Performance with different baseline models and the model with all extensions.

Dataset	F1 score				
	Avg	Lstm	Avg-t	Lstm-t	Allcomp-all sim-num-null-t
Amazon-google	0.66	0.81	0.865	0.82	0.84
Amazon-Walmart	0.849	0.855	0.875	0.82	0.879
dblp-scholar	0.97	0.975	0.975	0.98	0.99

**Figure 11:**
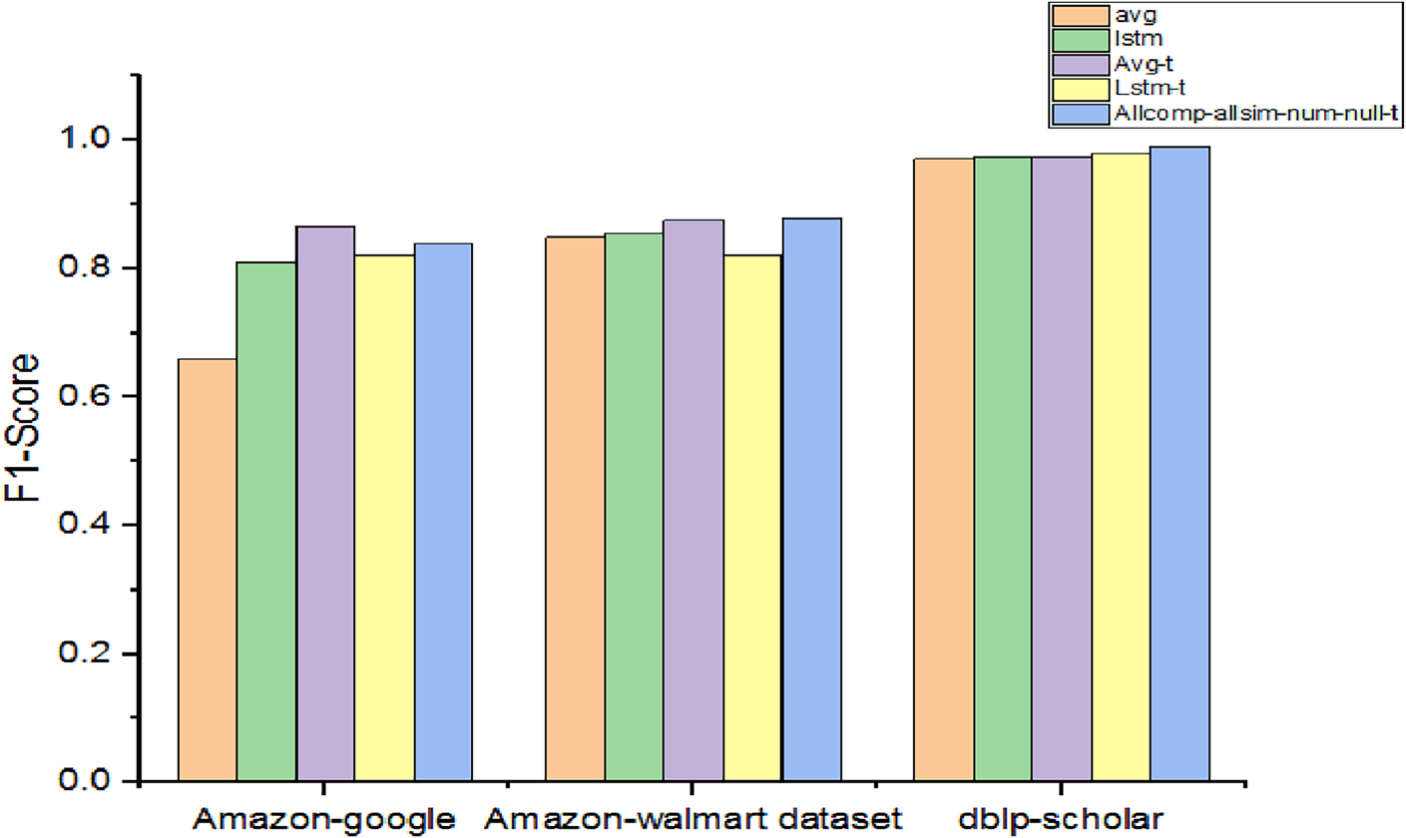
Performance comparison among baseline methods and methods with all extensions.

As presented in Fig. 11, the first four are baseline models and the last model is the model with all extensions. Both avg-t and lstm-t models performed better than their baseline counterparts due to the usage of trainable embedding. The model that is equipped with all extensions showed better performance with the highest F1-Score for all the datasets except the Amazon-Google dataset. However, in this case, Avg-t is the only method showing better performance over it.

As presented in Table 13, two models are compared for their performance. Out of them, avg-num-t supports numeric attributes also along with trainable embedding.

**Table 13:** Performance of two models.

Dataset	F1- score	
	Avg-t	Avg-num-t
Amazon-google	0.86	0.89
Amazon-Walmart dataset	0.875	0.91
dblp-scholar	0.97	0.975

**Figure 12:**
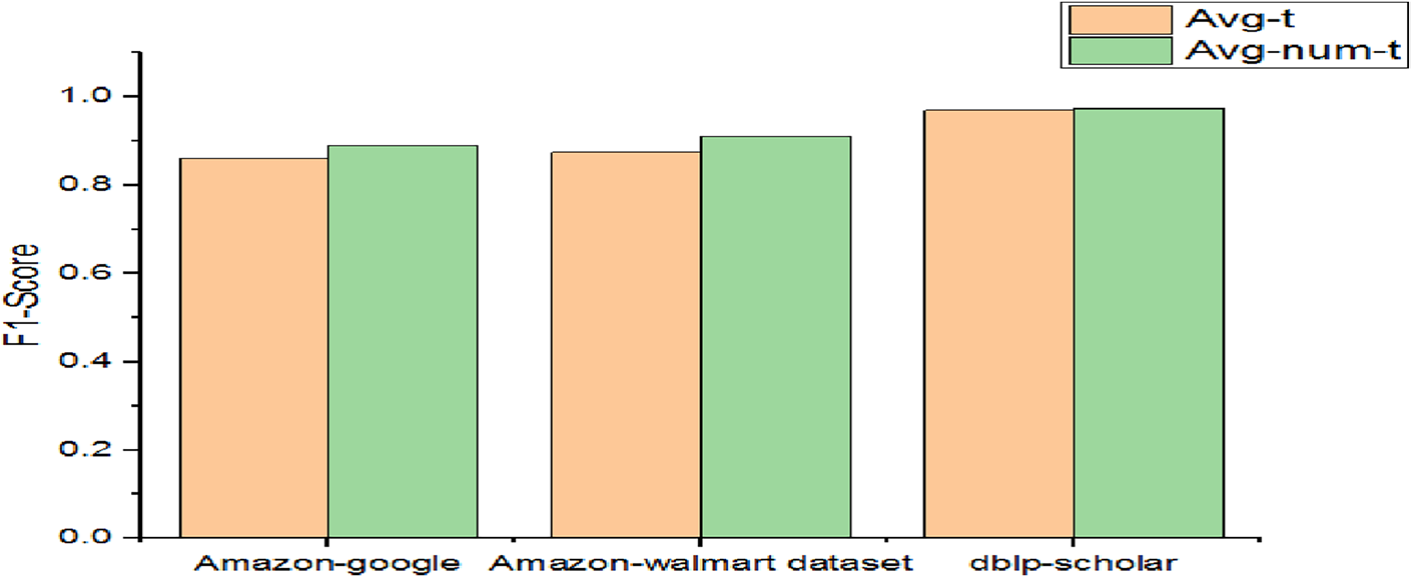
Performance comparison of two models.

As presented in Fig. 12, significant improvement is achieved by the model which supports both trainable embeddings and also numeric attributes. Since numeric attributes are not supported by the models in^6^, our contribution with extensions led to significant improvement in performance. Avg-t showed 86% F1-Score with the first dataset while avg-num-t showed 89% reflecting significant performance improvement. Similar kinds of observations are made between the two models for the second dataset. However, concerning the first dataset, the performance improvement achieved by avg-num-t is 0.5% only. From the empirical study and results, we summarize our findings here. The models with proposed extensions outperform baseline models associated with the reference model in^6^. Out of all baseline models avg-t showed better performance over other models except in the case of the dblp-scholar dataset. Its performance improvement is due to the support for trainable embeddings. Every model with extensions incorporated could provide better performance over the average model against all three datasets. The avg-num-t model is found to perform better than avg-t for all datasets. It is also observed that avg-t could perform better than the last variants for all databases.

The proposed model with all extensions provided in this paper is compared with state-of-the-art methods found in the literature in terms of F1-score as shown in Table 14.

**Table 14:** Performance comparison of the proposed model with state-of-the-art.

Dataset	F1- score		
	MDedup^48^	Hierarchical NN^49^	Proposed model (with all extensions)
Amazon-google	0.56	0.68	0.84
Amazon-Walmart dataset	0.49	0.58	0.879
dblp-scholar	0.83	0.90	0.99

**Figure 13:**
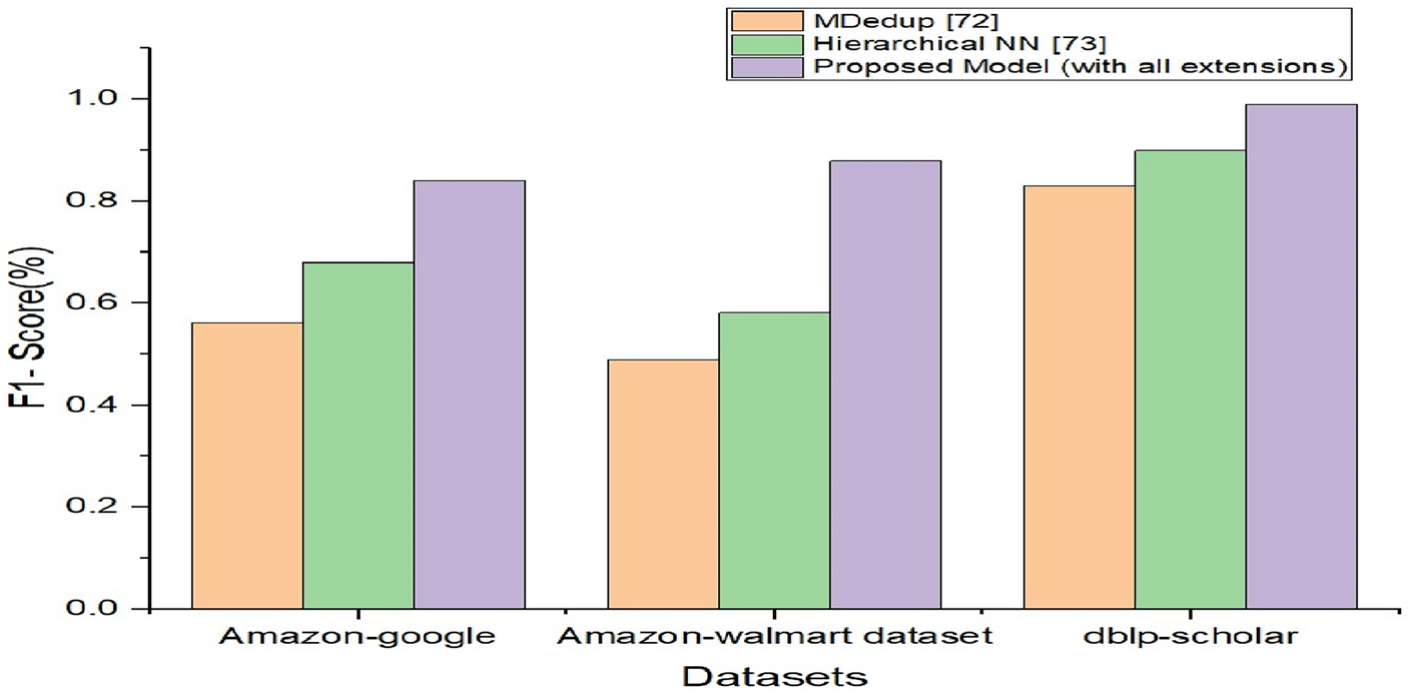
Performance comparison of the proposed model with existing methods.

As presented in Fig. 13, different deduplication models are compared against three data sets used in the empirical study in terms of the F1-score. Each model showed a different performance due to its underlying approach. MDedup method exhibited a 56% F1-score for the Amazon-Google dataset, 49% for the Amazon-Walmart dataset and 83% for the dblp-scholar dataset. The Hierarchical NN model showed a 68% F1-score with the Amazon-Google data set, 58% with the Amazon-Walmart dataset and 90% with the dblp-scholar dataset. The proposed model with all extensions could achieve an 84% F1-score with the Amazon-Google dataset, 87.90% with the Amazon-Walmart dataset and 99% F1-score with the dblp-scholar dataset. From the experimental results, it is observed that the proposed model outperforms the existing methods.

## Practical and managerial implications

Cloud computing infrastructure is widely used for storing and managing large volumes of data by enterprises in the real world. The data stored in cloud infrastructure may have duplicates that will impact storage efficiency and also energy efficiency in the cloud data centres. Therefore, deduplication plays a crucial role in improving infrastructure efficiency besides other indirect benefits to cloud service providers. Instead of heuristics-based approaches, with the emergence of Artificial Intelligence (AI), the learning-based approach has the potential to gain the required knowledge from time to time towards efficient detection of duplicate entities. For automatic record deduplication, several techniques came into existence. The proposed learning-based record deduplication methodology in this paper is found efficient due to various extensions made to the model. When compared with the state-of-the-art models, the proposed deep learning-based model could improve performance in the process of record deduplication. Therefore, the proposed methodology can have practical and managerial implications on the storage infrastructure and stakeholders in the real world. It can have possible implications on cloud infrastructure as it could reduce storage requirements due to efficient deduplication methodology. Since large volumes of data are stored and managed in the cloud, a small improvement in memory conservation leads to big results in terms of improving infrastructure efficiency, and energy efficiency and supporting service level agreements more efficiently. The proposed model when used in the cloud infrastructure, will have managerial implications as it can enable the infrastructure for automatic detection of duplicate records.

## Conclusion and future work

In this paper, we propose a framework known as Enhanced Deep Learning-based Record Deduplication (EDL-RD) for improving performance further. Towards this end, we exploited a variant of Long Short Term Memory (LSTM) along with various attribute compositions, similarity metrics, and numerical and null value resolution. We proposed an algorithm known as Efficient Learning based Record Deduplication (ELbRD). The algorithm extends the reference model with the aforementioned enhancements. Empirical study has revealed that the proposed framework with extensions outperforms existing methods. From the results, it is observed that deep learning is a powerful alternative for dealing with duplicates in voluminous data. The proposed model with all extensions could outperform all existing models with 84% F1-score with the Amazon-Google dataset, 87.90% with the Amazon-Walmart dataset and 99% F1-score using the dblp-scholar dataset. The proposed framework is not without limitations. It achieves attribute embeddings by using either the averaging method or LSTM. When the same LSTM is used across the attributes, it has issues in terms of power and expression. The rationale behind this is that for given tokens semantic meaning might differ based on the context. Therefore, exploiting different LSTMs for learning can help in dealing with attributes of different lengths. Another important problem identified is that the existing method has a drawback about sharing. Embedding layer sharing across the methods may impact performance due to overfitting. In future, we investigate on these two specific limitations of our framework EDL-RD.

## Data Availability

Data about this research is available with the corresponding author and it can be obtained by sending a request through an email.
